# 407. Prevalence of Post-COVID Sequelae Amongst Fully Vaccinated and Unvaccinated US Hospitalized Veterans

**DOI:** 10.1093/ofid/ofad500.477

**Published:** 2023-11-27

**Authors:** Sujee Jeyapalina, Guo Wei, Greg Stoddard, Aaron Millier, Jayant Agarwal

**Affiliations:** University of Utah, SALT LAKE CITY, Utah; University of Utah, SALT LAKE CITY, Utah; University of Utah, SALT LAKE CITY, Utah; University of Utah, SALT LAKE CITY, Utah; University of Utah, SALT LAKE CITY, Utah

## Abstract

**Background:**

Post-Acute Sequelae of COVID-19, also known as long COVID, refers to a wide range of ongoing health symptoms and illnesses that persist for weeks or months after the acute phase of the COVID-19 illness has resolved. Based on the overwhelming reports of multiple health symptoms, the ICD-10 code U09.9 became effective on October 1, 2021, for coding ongoing symptoms or health problems related to COVID-19, a range of prevalence for long-COVID is reported. Thus, we hypothesized that among veterans who experienced a COVID-19 infection that required hospitalization, with follow-up beginning with the first hospitalization and assigning vaccination status on first hospital admission date, subsequent long COVID prevalence would be greater in the unvaccinated.

**Methods:**

The deidentified veterans' data were accessed from the VA COVID-19 Shared Data Resources with local ethical approvals. The Veterans Affairs Informatics and Computing Infrastructure was used to extract patient health records for all U.S. veterans above the age of 18 who had tested positive for COVID-19. The primary outcome was the prevalence of reported U09.9 code after a single COVID-19 infection. The prevalence rates were calculated as the proportion of long-COVID within veterans who tested positive for COVID-19 disease between 10/01/2021 to 03/30/2023.

**Results:**

In total, 468,620 veterans who were older than 18 reported being positive for COVID-19 within the studied period. Also, 68,125 veterans had reported multiple COVID-19 infections and were excluded from the study. Within this cohort, 15,772 veterans had the U09.09 code assigned, indicating a long-COVID prevalence rate of 3.93%. Although no significant difference was found between the prevalence of long-COVID between the vaccinated (3.98%) and unvaccinated (3.87%) veterans, post-hospitalization, vaccinated veterans had a much-reduced prevalence rate (3.50%) than that of the unvaccinated group (8.78%). This trend was mirrored by the requirement for mechanical ventilation.

**Conclusion:**

Although the prevalence of long-COVID is 3.93% within the entire Veteran population, the prevalence of reported long-COVID was ∼three-fold greater in unvaccinated hospitalized veterans, with a ∼2.5-fold greater requirement for mechanical ventilation in the unvaccinated

**Disclosures:**

**All Authors**: No reported disclosures

